# Comparison of quality performance metrics in screening and surveillance colonoscopy: a single-center experience

**DOI:** 10.1016/j.igie.2024.08.007

**Published:** 2024-09-05

**Authors:** James Stephen Love, Michael Siegel, Meredith Yellen, Jeffrey Rebhun, Asim Shuja

**Affiliations:** 1 Department of Internal Medicine Chicago, University of Illinois at Chicago, Illinois, USA; 2 Department of Medicine, Division of Gastroenterology, University of Illinois at Chicago, Chicago, Illinois, USA; 3 Department of Medicine, Division of Gastroenterology and Hepatology, Oregon Health & Science University, Portland, Oregon, USA; 4 Division of Gastroenterology and Hepatology, University of Illinois at Chicago, Chicago, Illinois, USA

## Abstract

**Background and Aims:**

Screening colonoscopy guidelines recommend a minimum adenoma detection rate (ADR) of 35%. There are no established benchmarks for surveillance colonoscopies, and data surrounding the utility of other quality metrics are limited. We aimed to define the relationship between ADR and alternative quality measures in the setting of screening and surveillance colonoscopies and to determine whether validated screening quality benchmarks can be extrapolated to surveillance procedures.

**Methods:**

A retrospective review of outpatient screening and surveillance colonoscopies at a tertiary health center was performed. ADR, adenomas per colonoscopy, adenomas per positive participant, polyp detection rate, right-sided polyp detection rate, and colonoscopy withdrawal times (CWTs) were analyzed for screening and surveillance colonoscopies.

**Results:**

In total, 2646 procedures (1884 screening, 762 surveillance) were analyzed. Surveillance ADR (CADR) was higher than screening ADR (65.6% ± 0.02% vs 47.0% ± 0.01%; *P* < .001). All alternate quality measures except CWT were higher in surveillance procedures. Among surveillance procedures, there was a strong correlation between CADR and polyp detection rate (*r* = .956, *P* < .01) and right-sided polyp detection rate (r = .771, *P* = .003); correlations between CADR and other alternate quality measures were not significant.

**Conclusions:**

Colonoscopy quality measures were significantly higher in surveillance procedures compared with screening procedures despite similar CWTs. Higher benchmarks should be considered to ensure quality surveillance colonoscopies.

Colorectal cancer (CRC) is the third most common cause of cancer in the United States with an estimated cost of more than $17 billion per year.^1^^,^^2^ Screening colonoscopies allow for early detection and removal of precancerous colonic lesions, and they reduce the incidence of CRC.^1^^,^^3^ For these reasons, colonoscopy is heralded as the criterion standard CRC screening tool. To ensure high-quality colonoscopy performance, several quality indicators have been introduced. Adenoma detection rate (ADR), defined as the proportion of screening colonoscopies performed that detect at least 1 histologic colorectal adenoma or adenocarcinoma, is the most widely studied and validated quality indicator for screening colonoscopy.^4^ Initially, the ADR benchmark of >35% was considered adequate; however, recent trends in screening colonoscopies have shown significant increases in average ADR, estimated to be approximately 40%, suggesting the need to raise the ADR benchmark.^4^^,^^5^

There are several factors that may affect ADR for routine screening colonoscopies, such as endoscopist experience, patient sex, day of the week, and adequacy of bowel preparation.^6^^,^^7^ Consequently, adenoma detection is endoscopist dependent, with considerable variability between endoscopists.^8^ Further complicating this is the observation that high ADR does not always correlate with lower adenoma miss rates, suggesting that ADR as a sole indicator of colonoscopy quality performance is likely inadequate.^8^, ^9^, ^10^ In an attempt to reduce variation and provide additional information on endoscopist performance, several other quality indicators have been introduced, including adenomas per colonoscopy (APC), adenomas per positive participant (APP), polyp detection rate (PDR), colonoscopy withdrawal time (CWT), and right-sided polyp detection rate (RSP).^4^^,^^8^^,^^11^, ^12^, ^13^

Although these quality parameters have shown utility in distinguishing high-performing endoscopists beyond the standard ADR metric in screening colonoscopies, research regarding their role in surveillance procedures is limited, and there are no established benchmarks to ensure quality colonoscopy performance in this setting. The aim of the current study was to define the relationship between ADR and alternative quality indicators (APC, APP, PDR, CWT, and RSP) in the setting of screening and surveillance colonoscopies and to determine whether validated screening quality benchmarks can be extrapolated to surveillance procedures.

## Methods

### Study design

This retrospective observational study included all adult patients who underwent outpatient screening and surveillance colonoscopies from January 2015 to April 2020. Procedures were completed at a single university tertiary health center by board-certified gastroenterologists using standard adult colonoscopes. This study was approved by the Institutional Review Board at the University of Illinois Hospital and Health Sciences System in March 2020.

### Study population

The study population comprised 2646 patients undergoing outpatient screening and surveillance colonoscopies from January 2015 to April 2020. Patients with a history of inflammatory bowel disease, familial adenomatous polyposis syndrome, serrated polyposis syndrome, and CRC were excluded from the study. Subjects who underwent colonoscopy for positive fecal immunohistochemical testing and subjects who underwent colectomy for CRC were excluded from the study. Procedures with failed cecal intubation, inadequate bowel preparation, and diagnostic intentions were also excluded.

### Definition of variables and outcomes

ADR, APC, APP, PDR, RSP, and CWT were analyzed for screening and surveillance colonoscopies. Screening colonoscopy was defined as a colonoscopy performed on an asymptomatic patient with no history of colonoscopy or history of adenomatous polyps on previous colonoscopy. Surveillance colonoscopy was defined as a colonoscopy performed on a patient with history of adenomatous polyps on previous screening colonoscopy or CRC with complete endoscopic resection. ADR was defined as the number of colonoscopies performed that detect at least 1 histologic colorectal adenoma or adenocarcinoma divided by the number of colonoscopies. APC was defined as the number of detected adenomas divided by the total number of screening colonoscopies. APP was defined as the number of detected adenomas divided by the number of screening colonoscopies in which 1 or more adenomas are detected. PDR was defined as the number of colonoscopies in which at least 1 polyp was detected divided by the total number of colonoscopies. RSP was defined as the number of colonoscopies in which at least 1 polyp was detected in the right side of the colon divided by the total number of colonoscopies. CWT was defined as the time spent visualizing the colon from cecal intubation to withdrawal of the scope from the anus. To better estimate the time spent visualizing the colon, withdrawal times were corrected for by the total number of polyps resected respective of the individual procedure.

Bowel preparation was scored by using the Boston Bowel Preparation Scale. A procedure with a score <6 was deemed as having inadequate preparation.

### Statistical analysis

Categorical data are presented as percentages, and continuous data are presented as mean ± standard deviation. A χ^2^ analysis was performed to compare categorical variables. Normality tests were performed for each continuous variable, and Mann Whitney *U* or Kruskal-Wallis tests were performed to analyze continuous outcomes as appropriate. A 2-sided *P* value <.05 was used to determine statistical significance. Spearman rank correlations were performed to compare surrogate performance outcomes versus ADR; a strong correlation was defined by coefficient values between 0.7 and 1. Statistical analyses were performed by using SPSS Statistics for Macintosh version 27.0 (IBM SPSS Statistics, IBM Corporation, Armonk, NY, USA).

## Results

A total of 2646 procedures were included in our analysis. The average patient age for screening colonoscopies was 57.4 ± 7.9 years, and 44.5% were male (n = 838); the average patient age for surveillance colonoscopies was 62.2 ± 8.7 years, and 45.4% were male (n = 346) (Table 1). Patient demographic characteristics are described in Table 1. Screening colonoscopies accounted for 71.2% of included procedures (n = 1884), and surveillance colonoscopies accounted for the remaining 28.8% (n = 762).

**Table 1:** Baseline patient characteristics of the study population

Variable	Screening	Surveillance	Total	*P* value
Sex				.664
Female	1046 (55.5%)	416 (54.6%)	1462	
Male	838 (44.5%)	346 (45.4%)	1184	
Age, y	57.4 ± 7.9	62.2 ± 8.7		<.001
Race/ethnicity				.004
Black	1044 (55.4%)	432 (56.7%)	1476	
White	316 (16.8%)	155 (20.3%)	471	
Latino	344 (18.3%)	131 (17.2%)	475	
Asian	93 (4.9%)	15 (2.0%)	108	
Other	58 (3.1%)	21 (2.8%)	79	
Unknown	29 (1.5%)	8 (1.0%)	37	
Bowel preparation				<.001
Good	1164 (61.8%)	570 (74.8%)	1734	
Excellent	720 (38.2%)	192 (25.2%)	912	

Values are mean ± standard deviation.

Quality indicators for screening and surveillance colonoscopies are summarized in Table 2. Surveillance ADR (CADR) was significantly higher than screening ADR (SADR) (65.6% ± .02% vs 47.0% ± .01%; *P* < .001). All alternate quality measures, including ADR, APC, PDR, APP, and RSP, were significantly higher in surveillance procedures, with the exception of CWT. Among surveillance procedures, there was a strong correlation between CADR and PDR (*r* = .956, *P* < .01), as well as RSP (r = .771, *P* = .003); correlations between CADR and other alternate quality measures, including APP and APC, were not statistically significant (Table 3).

**Table 2:** Association of quality parameters between screening and surveillance procedures

Quality parameter	Screening (n = 1884)	Surveillance (n = 762)	*P* value
ADR	47.0 ± .01	65.6 ± .02	<.001
APC	1.60 ± .04	1.81 ± .07	<.001
APP	2.07 ± .06	2.30 ± .08	.012
PDR	64.7% ± .01	77.7 ± .02	<.001
RSP	45.3% ± .01	63.3% ± .02	<.001
CWT	15.8 ± 9.2	16.5 ± 9.7	.079

Values are mean ± standard deviation.

*ADR*, Adenoma detection rate; *APC*, adenoma per colonoscopy; *APP*, adenoma per positive participant; *PDR*, polyp detection rate; *RSP*, right-sided polyp detection rate; *CWT*, colonoscopy withdrawal time.

**Table 3:** Quality parameters and their association with ADR in surveillance procedures

Quality parameter	Spearman rank correlation (*r*)	*P* value
APC	.699	.011
APP	.21	.513
PDR	.956	<.001
RSP	.771	.003
CWT	–.049	.88

*ADR*, Adenoma detection rate; *APC*, adenoma per colonoscopy; *APP*, adenoma per positive participant; *PDR*, polyp detection rate; *RSP*, right-sided polyp detection rate; *CWT*, colonoscopy withdrawal time.

## Discussion

The current study evaluated quality performance metrics in outpatient screening and surveillance colonoscopies at a single-center tertiary health center. Overall, we found that all quality measures were significantly higher in surveillance procedures compared with screening procedures despite similar CWT, suggesting that separate ADR targets for surveillance and screening procedures should be established to ensure quality performance. This is inconsistent with previously reported data, wherein similar rates of ADR and other quality metrics were found between screening and surveillance procedures.^14^

The current study data also suggest that colonoscopy quality measures were significantly higher in surveillance procedures versus screening procedures despite similar CWTs. This is inconsistent with earlier similar studies, in which CADR and SADR were not significantly different, and far exceeds previously reported CADR rates.^14^, ^15^, ^16^ The SADR among our cohort was approximately 47%, which exceeds the recommended minimum benchmark of 35%. As mentioned, SADR can vary widely depending on certain patient populations and practice settings; however, according to a large national quality benchmarking registry, recent trends in screening colonoscopy performance suggest that the SADR achieved in most practices is closer to 35% to 40%, which is similar to the SADR observed in our tertiary health center.^5^^,^^17^ However, because the entry age for CRC screening has recently been lowered to include those ≥45 years and older, and while alternatives such as the fecal immunochemical test are becoming increasingly popular, it is reasonable to suspect that there will be larger demand for surveillance colonoscopies compared with screening colonoscopies in the near future.^18^^,^^19^ We should thus anticipate a larger volume of surveillance procedures and establish appropriate benchmarks to ensure quality surveillance colonoscopy performance and to improve detection rates of interval CRC.

In addition, we aimed to directly compare quality measures across surveillance colonoscopies and explore their correlations. In a previous study, it was found that all quality parameters except CWT correlate strongly with ADR in screening colonoscopies.^6^ Interestingly, in the setting of surveillance colonoscopies, we found that only PDR and RSP were strongly associated with CADR. This novel finding suggests that APP and APC may provide additional information regarding surveillance endoscopist performance, although further studies are needed to strengthen this association.

The current study is not devoid of limitations. The primary aim of the study was to assess overall quality measures such as ADR, APC, APP, PDR, RSP, and CWT across screening and surveillance colonoscopies completed at a single tertiary health center by multiple board-certified gastroenterologists. The study was not specifically designed to evaluate individual endoscopist performance; thus, the data collected and the analytical methods used were tailored to broader comparisons rather than individual assessments. Furthermore, given that this is a single-center study, it is difficult to assess the generalizability and implications of these results at different practices. In addition, endoscopists were not blinded during this study, and each endoscopist was aware of each patient’s history and reason for colonoscopy. The heightened awareness of reducing interval CRC incidence during surveillance procedures may have resulted in improved colonoscopy performance and a drastically higher CADR, as seen in this study, although the mechanism of this finding is unclear as there was no significant difference in CWT between surveillance and screening procedures. Due to these limitations, conducting a reliable and valid subgroup analysis of individual endoscopists' performance within the scope of this study was not feasible.

This was a retrospective study in which data collection relied on existing medical records, which may not include all necessary details for a comprehensive subgroup analysis. Descriptions for surveillance colonoscopy were often limited, and complete patient histories were not consistently available. Thus, it was not possible to compare quality metrics between high-risk and low-risk subgroups based on index colonoscopy before surveillance procedures or family histories of CRC.

Few studies have directly compared the utility of ADR and other quality indicators in screening and surveillance procedures. Patients with a history of colorectal polyps are at high risk for polyp recurrence and progression to CRC, yet there are no established benchmarks for quality indicators for surveillance procedures.^14^^,^^16^ Our findings directly aid in closing this gap in our scientific literature and are strengthened by a large sample size of >2600 colonoscopies performed by 29 endoscopists at a major academic tertiary center with a diverse patient population.

In conclusion, all quality indicators in this study were significantly higher in surveillance procedures compared with screening procedures despite similar CWTs. To our knowledge, this study is the first showing the need for higher-quality benchmarks for surveillance colonoscopies relative to screening colonoscopies. This study confirms recent trends in screening colonoscopies, echoing the need for a higher SADR benchmark and reporting a positive correlation between SADR and all other quality metrics in screening procedures. Furthermore, among surveillance procedures, CADR did not correlate with APP and APC, suggesting these quality indicators may provide additional information regarding surveillance endoscopist performance. The inconsistency with previous studies is interesting and may be attributed to several factors, including differences in study populations, endoscopist techniques, and definitions of quality metrics. In light of these findings and inconsistencies with previous studies, to establish definitive quality benchmarks for surveillance colonoscopies and to ensure endoscopist quality performance in a larger variety of settings, we recommend that similar prospective studies be conducted in multiple geographically diverse settings with a focus on individual endoscopist performance and controlled data collection in order to perform subgroup analyses based on endoscopic expertise and patient risk factors (eg, family history of CRC).

## Disclosure

All authors disclosed no financial relationships.
